# Organizational factors associated with target sedation on the first 48 h of mechanical ventilation: an analysis of checklist-ICU database

**DOI:** 10.1186/s13054-019-2323-y

**Published:** 2019-01-29

**Authors:** Antonio Paulo Nassar, Fernando G. Zampieri, Jorge I. Salluh, Fernando A. Bozza, Flávia Ribeiro Machado, Helio Penna Guimarães, Lucas P. Damiani, Alexandre Biasi Cavalcanti

**Affiliations:** 1 Intensive Care Unit and Postgraduate Program, A.C. Camargo Cancer Center, São Paulo, Brazil; 2 Research Institute, HCor-Hospital do Coração, São Paulo, Brazil; 3 Hospital Alemão Oswaldo Cruz, São Paulo, Brazil; 4 Graduate Program in Translational Medicine and Department of Critical Care, D’Or Institute for Research and Education, Rio De Janeiro, Brazil; 5 Programa de Pós-Graduação em Clinica médica, Universidade Federal do Rio de Janeiro, Rio de Janeiro, Brazil; 6 Instituto Nacional de Infectologia Evandro Chagas, Fundação Oswaldo Cruz (FIOCRUZ), Instituto D’Or de Pesquisa e Ensino (IDOR), Rio de Janeiro, Brazil; 7 Anesthesiology, Pain and Intensive Care Department, Federal University of São Paulo, São Paulo, Brazil; 8 Federal univeristy of São Paulo, São Paulo, Brazil

**Keywords:** Conscious sedation, Critical care, Deep sedation, Mechanical ventilation, Outcome and process assessment

## Abstract

**Background:**

Although light sedation levels are associated with several beneficial outcomes for critically ill patients on mechanical ventilation, the majority of patients are still deeply sedated. Organizational factors may play a role on adherence to light sedation levels. We aimed to identify organizational factors associated with a moderate to light sedation target on the first 48 h of mechanical ventilation, as well as the association between early achievement of within-target sedation and mortality.

**Methods:**

This study is a secondary analysis of a multicenter two-phase study (prospective cohort followed by a cluster-randomized controlled trial) performed in 118 Brazilian ICUs. We included all critically ill patients who were on mechanical ventilation 48 h after ICU admission.

A moderate to light level of sedation or being alert and calm (i.e., the Richmond Agitation-Sedation Scale of − 3 to 0) was the target for all patients on mechanical ventilation during the study period. We collected data on the type of hospital (public, private, profit and private, nonprofit), hospital teaching status, nursing and physician staffing, and presence of sedation, analgesia, and weaning protocols. We used multivariate random-effects regression with ICU and study phase as random-effects and correction for patients’ Simplified Acute Physiology Score 3 and Sequential Organ Failure Assessment. We also performed a mediation analysis to explore whether sedation level was just a mediator of the association between organizational factors and mortality.

**Results:**

We included 5719 patients. Only 1710 (29.9%) were on target sedation levels on day 2. Board-certified intensivists on the morning and afternoon shifts were associated with an adequate sedation level on day 2 (OR = 2.43; CI 95%, 1.09–5.38). Target sedation levels were associated with reduced hospital mortality (OR = 0.63; CI 95%, 0.55–0.72). Mediation analysis also suggested such an association, but did not suggest a relationship between the physician staffing model and hospital mortality.

**Conclusions:**

Board-certified intensivists on morning and afternoon shifts were associated with an increased number of patients achieving lighter sedation goals. These findings reinforce the importance of organizational factors, such as intensivists’ presence, as a modifiable quality improvement target.

**Electronic supplementary material:**

The online version of this article (10.1186/s13054-019-2323-y) contains supplementary material, which is available to authorized users.

## Background

The burden of oversedation has been recognized for many years [1], even after adjustment for potential confounders. In addition, strategies aiming at light sedation levels have consistently demonstrated efficacy in reducing time on mechanical ventilation, intensive care unit (ICU), and hospital length of stay [2, 3]. More recently, even deep sedation levels on the first 48 h of mechanical ventilation were associated with negative outcomes, including increased mortality [4, 5].

Despite the vast number of studies showing light sedation levels associated with positive outcomes in patients on mechanical ventilation, its adoption is far from being widespread. Observational studies suggest up to two thirds of patients on mechanical ventilation are deeply sedated [6–10]. In the checklist-ICU study, even if the intervention led to a higher compliance to predefined goals as compared with the control group, only 40.5% of patients on mechanical ventilation in the intervention group achieved the target sedation level [11].

Organizational factors and processes of care are associated with improved outcomes in critically ill patients such as high-intensity staffing ICUs [12, 13], continuity of care [14], multidisciplinary rounds [15], and adoption of protocols [16]. ICU context factors, such as safety culture, lack of leadership, and lack of interprofessional team support, may play a role as barriers to an effective implementation of a bundle of awaking and breathing coordination, delirium, and early mobilization [17]. However, no study evaluated what organizational factors were related to achieving sedation goals.

Therefore, we aimed to identify organizational factors that were associated with moderate to light sedation levels on the first 48 h of mechanical ventilation after ICU admission by performing a secondary analysis of a large quality improvement cluster-randomized RCT [11]. Additionally, we aimed to assess the association of early moderate to light sedation levels with hospital mortality.

## Methods

### Study design and patients

A detailed description of the checklist-ICU study has been published previously [11, 18]. In brief, the checklist-ICU study was conducted in two phases. In phase 1, we assessed organizational factors and clinical outcomes in 118 Brazilian adult ICUs from August 2013 to March 2014. In phase 2, these ICUs were randomized to a quality improvement intervention or to usual care from April to November 2014. The intervention consisted of a checklist and discussion of goals of care during daily multidisciplinary rounds, with follow-up clinician prompting to ensure checklist adherence and goals of care for all patients during their ICU length of stay. The checklist assessed prevention and management of common ICU problems (venous thromboembolism, ventilator-associated pneumonia, central line-associated bloodstream, urinary tract infection, nutritional, analgesia and sedation goals, adherence to low-tidal volume (≤ 8 ml/kg) ventilation, assessment of readiness for extubation, detection of sepsis and acute respiratory distress syndrome, and antibiotic initiation and stewardship). Specifically, for sedation goals, it was recommended that all patients on mechanical ventilation should have a target sedation level of − 3 to 0 in the Richmond Agitation-Sedation Scale (RASS). Other care processes, with the exception of the checklist, were unchanged between phases 1 and 2.

The ethics committees of all institutions approved the study. The funding source had no role in the analysis or publication decisions.

For this secondary analysis, we included patients from both study phases. Only patients 18 years or older who were on mechanical ventilation 48 h after ICU admission were included. We excluded patients with suspected or confirmed brain death, admitted with comfort only measures, and with a high probability of dying before 72 h of ICU stay.

### Data collection

We gathered baseline information on age, sex, reason for admission, type of admission (clinical, elective or urgent surgery), illness severity [Simplified Acute Physiology Score (SAPS) 3 and Sequential Organ Failure Assessment (SOFA)], and in what phase of study the patient was included. We also collected ICU and hospital mortality and ICU and hospital length of stay.

Sedation was assessed using RASS at the multidisciplinary round on days 2, 5, 8, 11, 14, and 17 after ICU admission. A moderate to light level of sedation or being alert and calm (i.e., RASS − 3 to 0) was the target for all patients on mechanical ventilation during the study period. Patients with RASS levels ≤ − 4 and ≥ 1 were considered deeply sedated or agitated, respectively. For the purpose of the present study, we considered an “adequate” level of sedation if the patient was on target at each evaluation and “inadequate” if the patient was either deeply sedated or agitated.

We recorded the following organizational data related to the process of care of each participating ICU:

- Type of hospital (public, private, profit and private, nonprofit);
- Teaching status (whether the hospital is university-affiliated or not);
- Presence of sedation, analgesia, and mechanical ventilation weaning protocols;
- Presence of board-certified ICU nurse coordination;
- Presence of at least one nurse technician for each two patients in all shifts; and
- Presence of a board-certified intensivist on the morning and afternoon shifts (no; yes, but only in one shift; or yes, in both).

We recorded the latter information because in Brazil, all ICUs must have dedicated physicians 24/7, but these physicians may or may not be board-certified intensivists. However, a board-certified intensivist on the morning (usually 7 am to 1 pm) and afternoon (usually 1 pm to 7 pm) shifts is required by regulatory agencies in Brazil. The most common physician staffing model in Brazil is one composed by a non-intensivist physician for each 10 ICU beds in a 12-h shift (7 am to 7 pm) and an intensivist who may be present on the morning, afternoon, or both shifts. The intensivist can either lead multidisciplinary rounds (usually on the morning) or rounds with the other physicians. A board-certified intensivist is one who completed his/her 2-year training in critical care medicine. In Brazil, when the study was performed, physicians could apply for a critical care fellowship program after accomplishment of a residency program in internal medicine, general surgery, or anesthesiology.

### Outcomes

The primary outcome of this study was the proportion of patients on a moderate to light level of sedation or being alert and calm (RASS − 3 to 0) on day 2. The secondary outcomes were the proportion of patients on the target sedation level on days 5, 8, 11, 14, and 17.

### Statistical analysis

Continuous variables are presented as medians and interquartile ranges and compared with the Mann-Whitney test. Categorical variables are presented as absolute numbers and percentage and compared with the chi-square test or Fisher’s exact test, as appropriate. The number of patients on adequate levels of sedation is presented as percentage and 95% confidence intervals (CI), adjusted for SAPS 3 and SOFA.

We investigated the association between adequate sedation levels and organizational factors first adjusting for patients’ SAPS 3 and SOFA using a multivariate random-effects logistic regression with ICU and study phase as random-effects (added as random intercepts in the model) and then including variables related to organizational factors. The variables included in the regression were defined a priori (type of hospital, teaching status hospital, presence of sedation, analgesia and/or mechanical ventilation weaning protocols, board-certified ICU nurse coordination, nurse technician to patient ratio ≤ 1:2 in all shifts, and presence of board-certified intensivists on the morning and afternoon shifts). We calculated the generalized variance-inflation factor (GVIF) to detect multicollinearity. To make GVIFs comparable across dimensions, we also calculated GVIF^(1/2 × df), in which df are degrees of freedom of the variable [19]. If GVIF^(1/2 × df) was higher than 2, it would be considered an indication of multicollinearity, and then only the most clinical relevant variable would be included in the model.

We also assessed the association between the sedation level on day 2 and hospital mortality with a random-effects logistic regression model, adjusted for SAPS 3 and SOFA at the patient level, and with ICU and study phase as random-effects. We performed a mediation analysis to assess whether the sedation level was only a mediator of an effect of organizational factors on mortality.

All analyses were performed with R project version 3.4 with RStudio Version 1.1.456.

## Results

A total of 13,638 patients were included in the original study. We included 5719 patients from 118 ICUs who were on mechanical ventilation on day 2 after ICU admission, 2783 in the observational phase and 2936 in the intervention phase. Most admissions were medical, and the main reasons for ICU admission were acute respiratory failure and sepsis. RASS levels were available for all patients on days 2, 5, 8, 11, 14, and 17. Only 1710 (29.9%) patients were at a RASS level of − 3 to 0 on the first 48 h of mechanical ventilation. Most patients (*n* = 3534, 61.8%) were deeply sedated. Only 475 (8.3%) were agitated.

Patients on adequate sedation levels were more frequently admitted for acute respiratory failure and had lower SAPS 3 and SOFA scores. Unadjusted ICU and hospital mortality were lower for patients with the target adequate sedation level. ICU and hospital length of stay were increased for patients with inadequate sedation levels (Table 1).

**Table 1:** Patients’ characteristics

	Adequate sedation level (*n* = 1710)	Inadequate sedation level (*n* = 4009)	*p*
Male sex, *N* (%)	946 (55.3)	2332 (58.2)	0.05
Age, median (IQR), years	61.4 (45.6–74.3)	60.6 (43.0–73.8)	0.08
Type of admission, *N* (%)			0.05
Medical	1280 (74.9)	3046 (76.0)	
Elective surgery	132 (7.7)	239 (6.0)	
Urgent surgery	298 (17.4)	724 (18.1)	
Reason for ICU admission, *N* (%)			< 0.01
Acute respiratory failure	466 (27.3)	857 (21.4)	
Sepsis	293 (17.1)	770 (19.2)	
Neurological disorders	204 (11.9)	612 (15.3)	
Cardiovascular disorders	74 (4.3)	163 (4.1)	
Comorbidities, *N* (%)			
Cancer	139 (8.1)	305 (7.6)	0.50
Heart failure	97 (5.7)	245 (6.1)	0.52
Cirrhosis	35 (2.0)	118 (2.9)	0.05
AIDS	73 (4.3)	192 (4.8)	0.39
Study phase, *N* (%)			0.33
Observational	861 (50.4)	2075 (51.8)	
Interventional	849 (49.6)	1934 (49.2)	
SAPS 3, median (IQR)	60 (48–71)	63 (51–75)	< 0.01
SOFA, median (IQR)	6 (3–8)	7 (4–9)	< 0.01
Outcomes			
ICU mortality, *N* (%)	635 (37.1)	1948 (48.6)	< 0.01
ICU length of stay median (IQR)	11 (6–18)	12 (7–19)	< 0.01
Hospital mortality, *N* (%)	784 (45.8)	2284 (57.0)	< 0.01
Hospital length of stay median (IQR)	25 (13–45)	23 (12–43)	< 0.01

*AIDS* acute acquired immunodeficiency syndrome, *ICU* intensive care unit, *SAPS* Simplified Acute Physiology Score, *SOFA* Sequential Organ Failure Assessment

**Fig. 1:**
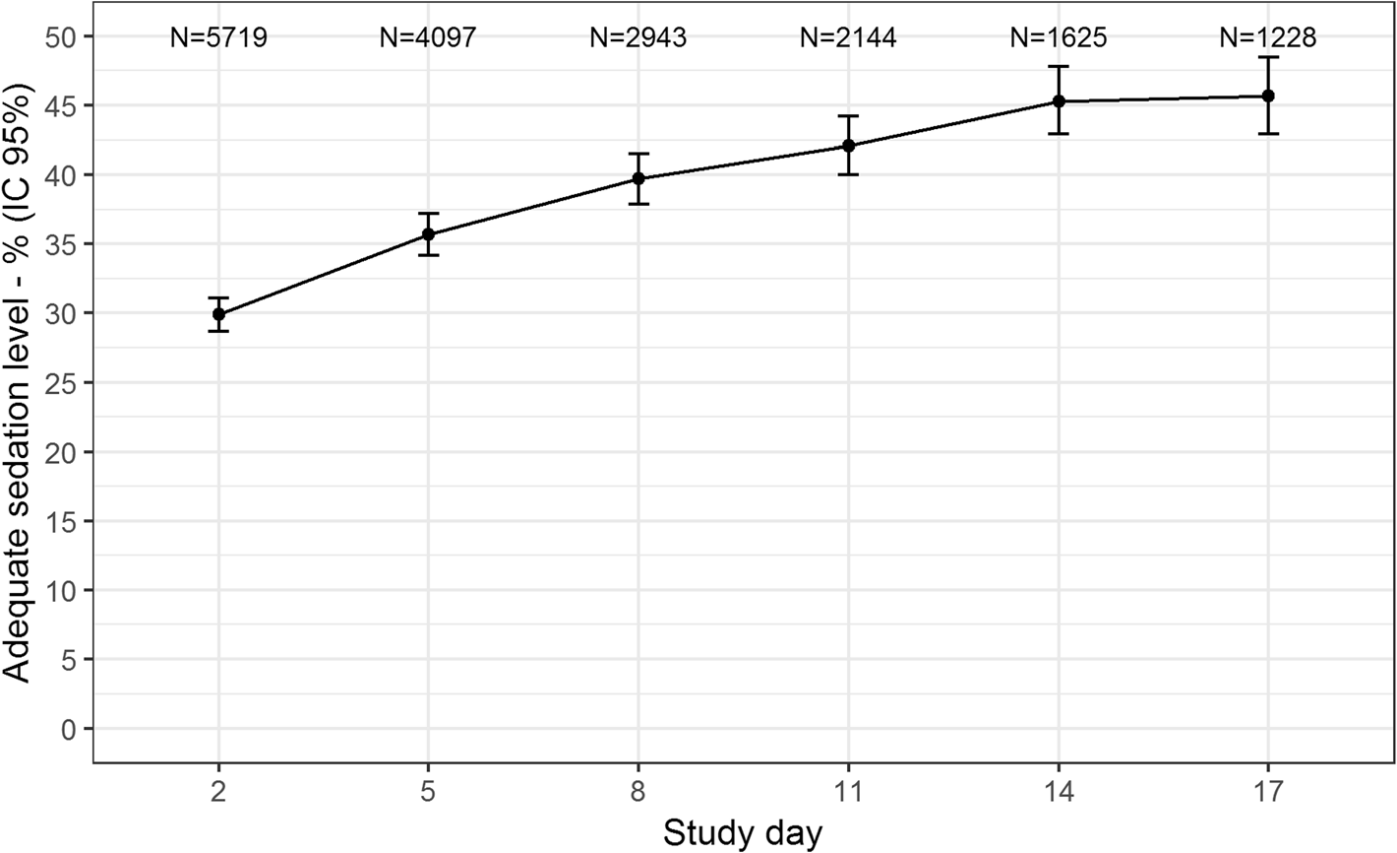
Proportion of patients on adequate sedation levels (i.e., RASS − 3 to 0) on each study assessment day

The proportion of patients on adequate sedation levels increased during observation days from 29.9%(CI 95%, 28.7–31.1) on day 2 reaching a maximum of only 45.7%(CI 95%, 42.9–48.5) on day 17 (Fig. 1).

Regarding the organizational factors, having a board-certified intensivist on both the morning and afternoon shifts was the sole factor associated with patients on mechanical ventilation being at a light to moderate sedation level (OR = 2.43; CI 95%, 1.09–5.38) (Table 2). There was no multicollinearity among the included variables (Additional file 1: Table S1).

**Table 2:** Organizational factors and the target sedation level on day 2

Organizational factor	Adequate sedation level/*N* (%)	Univariate analysis [OR (CI 95%)]	Multivariate analysis [OR (CI 95%)]
Type of hospital			
Private nonprofit	446/1455 (30.7)	Ref	Ref
Private profit	271/787 (34.4)	0.92 (0.54–1.57)	0.99 (0.57–1.70)
Public	992/3447 (28.5)	0.88 (0.56–1.37)	0.83 (0.54–1.28)
Teaching status			
No	1102/3466 (31.8)	Ref	
Yes	607/2253 (26.9)	1.04 (0.70–1.55)	1.02 (0.67–1.57)
Sedation protocol			
No	966/3031 (31.9)	Ref	Ref
Yes	743/2688 (27.6)	0.76 (0.52–1.10)	0.69 (0.36–1.32)
Analgesia protocol			
No	1069/3366 (31.8)	Ref	Ref
Yes	640/2353 (27.2)	0.80 (0.55–1.18)	1.19 (0.60–2.35)
Weaning protocol			
No	673/2007 (33.5)	Ref	Ref
Yes	1036/3712 (27.9)	0.77 (0.52–1.14)	0.78 (0.50–1.12)
Board-certified ICU chief nurse			
No	611/1924 (31.8)	Ref	Ref
Not available	151/334 (45.2)	2.03 (0.75–5.49)	2.10 (0.79–5.58)
Yes	947/3461 (27.4)	0.92 (0.61–1.37)	0.96 (0.65–1.43)
Nurse technician to patient ratio ≤ 1:2 in all shifts			
No	126/424 (29.7)	Ref	Ref
Yes	1583/5295 (29.9)	1.28 (0.64–2.53)	1.33 (0.68–2.61)
Board-certified intensivist on morning and afternoon shifts			
No	48/184 (26.1)	Ref	Ref
Only in one shift	608/2264 (26.9)	1.72 (0.76–3.91)	1.70 (0.75–3,87)
Yes, in both	1009/3084 (32.7)	2.26 (1.02–5.03)	2.43 (1.09–5.38)

A light to moderate sedation level on day 2 was associated with reduced hospital mortality when adjusted for SAPS 3 and SOFA (OR = 0.63; CI 95%, 0.55–0.72). The mediation model suggested that an adequate sedation level was significantly associated with hospital mortality (average causal mediation effect: *b* = − 0.0061; CI 95% − 0.0061 to < − 0.0001), but there was no direct effect of the physician staffing model (average direct effect: *b* = 0.0226; CI 95% − 0.0043 to 0.05000) on mortality (Additional file 1: Figure S1).

## Discussion

Our study suggested that having a board-certified intensivist on the morning and afternoon shifts was the only organizational factor associated with achieving target sedation levels in patients on mechanical ventilation 48 h after ICU admission. Additionally, we demonstrated that light and moderate sedation levels are associated with reduced hospital mortality. Interestingly, the number of patients on target sedation levels increased as long as ventilation time increased.

Daytime high-intensity staffing is associated with improved outcomes, such as mortality, and reduced time on mechanical ventilation and ICU length of stay [12, 20, 21]. We can postulate that achievement of light sedation levels on the first 48 h of mechanical ventilation may be one possible mechanism of improved outcomes in high-intensity staffing levels, since physician staffing did not have any direct effect on mortality in mediation analysis. Additionally, despite protocols were previously associated with improved outcomes in critically ill patients [16], it seems it is not enough just to have them in an ICU, since sedation protocols did not decrease time on mechanical ventilation in some specific settings [22, 23]. Probably, jointly management by different professionals seems to be of paramount importance for protocols to achieve their goals [16]. Our study suggests that intensivists may have an important role in lighter sedation targets. It is possible that intensivists may be more aware of the importance of light sedation goals than non-intensivists. The presence of more intensivists in an ICU may, hence, ensure that this target will be pursued with more determination. Therefore, a high-intensity staffing model may be part of a system of care, which then leads to improved outcomes [24]. This is an important finding as it represents a potentially modifiable factor. However, the shortage of intensivists in low- and middle-income countries [25, 26] precludes a wider adoption of a daytime high-intensivists staffing model. It is unknown whether other models, such as telemedicine, may increase adherence to lighter sedation levels.

We observed a positive association between early light and moderate sedation levels and reduced mortality, adjusting for patient severity. This association had already been demonstrated in previous studies [7, 8, 27, 28] and confirmed in a meta-analysis [4]. However, this is the largest cohort to show this relation ever. Although not new, these findings reinforce the need to reach light sedation levels from the beginning of mechanical ventilation.

The majority of patients (61.8%) were deeply sedated in the first 48 h of mechanical ventilation in our study. This result is similar to that from other studies that addressed the impact of early sedation on mortality [7, 8], but represents an opportunity for improvement since deep sedation may play a role in the high mortality observed in critically ill patients on mechanical ventilation in Brazil [29]. As expected, a higher proportion of patients were at light and moderate sedation levels as the mechanical ventilation time increased. We believe there are two hypotheses for this finding. First, there is a generalized perception that acute severely ill patients need to be deep sedated, since most studies addressing strategies of light sedation included patients only after 48 h of mechanical ventilation [30, 31], and performing a tracheostomy reduces the use of sedatives [32]. Unfortunately, we did not collect any data on severity on subsequent days after ICU admission neither on tracheostomy in the original study to address these hypotheses.

Our study has some limitations. First, it is a secondary analysis of a study designed to evaluate the effectiveness of a multifaceted quality improvement strategy including a checklist in ICU. Thus, some important data regarding sedation practices, mechanical ventilation, and patients’ severity are missing (for example, type and dosage of sedatives used, more frequent RASS assessments, a daily SOFA, delirium assessment, and number of tracheostomies performed). Second, it was not possible, from our database, to identify if a deep sedation level was secondary to sedatives or to non-modifiable causes, such as an acute encephalopathy. Third, we also do not have data regarding clinical pharmacists, whose interventions on sedation may be associated with decreased mechanical ventilation times [33]. These missing data and other potential confounders may have affected our results. Fourth, all included ICUs are from Brazil. It is widely known that organizational factors and process of care hugely differ worldwide. Although daytime high-intensity staffing may be an outdated problem in high-income countries, only 54% of patients of the present study were admitted to ICUs with board-certified intensivists on the morning and afternoon shifts. Low-income countries have lower percentages of board-certified intensivists in ICU compared to high-income countries [16, 26].

## Conclusions

Having board-certified intensivists on the morning and afternoon shifts was associated with an increased number of patients achieving lighter sedation goals. Lighter sedation goals are associated with decreased hospital mortality when adjusted for patients’ severity. Light sedation was more common as patients stay for more days on mechanical ventilation. These findings reinforce the importance of aiming a sedation level targeting an alert and calm patient early on mechanical ventilation and shed light on organizational factors that may increase adherence to this aim.

## Additional file

Additional file 1: **Table S1.** Generalized variance-inflation factor (GVIF) of the variables included in the logistic regression model. Df: degrees of freedom. GVIF: Generalized variance-inflation factor. SAPS: Simplified Acute Physiology Score. SOFA: Sequential Organic Failure Assessment. Figure S1**.** Mediation model. The mediation analysis suggested that sedation level was associated with hospital mortality [average causal mediation (ACME) effect: *b*=−0.0061; CI95% −0.0061 to <−0.0001], but board certified intensivists on morning and afternoon shifts were not [average direct effect (ADE): *b*=0.0226; CI95% −0.0043 to 0.05000]. (DOCX 28 kb)

## Abbreviations

CI: Confidence interval
GVIF: Generalize variance-inflation factor
ICU: Intensive care unit
RASS: Richmond Agitation-Sedation Scale
SAPS: Simplified Acute Physiology Score
SOFA: Sequential Organ Failure Assessment
