# Supplementary material for: Organizational factors associated with target sedation on the first 48 h of mechanical ventilation: an analysis of checklist-ICU database
Source: Crit Care. 2019 Jan 29;23:34. doi: 10.1186/s13054-019-2323-y (PMC6352335; doi:10.1186/s13054-019-2323-y)
Supplement: Supplementary file 1 — Table S1. Generalized variance-inflation factor (GVIF) of the variables included in the logistic regression model. Df: degrees of freedom. GVIF: Generalized variance-inflation factor. SAPS: Simplified Acute Physiology Score. SOFA: Sequential Organic Failure Assessment. Figure S1. Mediation model. The mediation analysis suggested that sedation level was associated with hospital mortality [average causal mediation (ACME) effect: b=−0.0061; CI95% −0.0061 to <−0.0001], but board certified intensivists on morning and afternoon shifts were not [average direct effect (ADE): b=0.0226; CI95% −0.0043 to 0.05000]. (DOCX 28 kb) [file 13054_2019_2323_MOESM1_ESM.docx]

Table S1. Generalized variance-inflation factor (GVIF) of the variables included in the logistic regression model.

| **Variable** | **GVIF** | **GVIF^(1/2*df)** |
| --- | --- | --- |
| SAPS 3 | 1.39 | 1.18 |
| SOFA | 1.40 | 1.18 |
| Type of hospital | 1.35 | 1.08 |
| Teaching status | 1.30 | 1.14 |
| Sedation protocol | 3.35 | 1.83 |
| Analgesia protocol | 3.57 | 1.89 |
| Weaning protocol | 1.40 | 1.18 |
| Board-certified ICU nurse coordination | 1.12 | 1.03 |
| Nurse technician to patient ratio ≤ 1:2 in all shifts | 1.18 | 1.04 |
| Board-certified intensivist on morning and afternoon shifts | 1.08 | 1.04 |

Df: degrees-of-freedom. GIVF: Generalized Variance-Inflation Factor. SAPS: Simplified Acute Physiology Score. SOFA: Sequential Organic Failure Assessment.

Figure S1. Mediation model
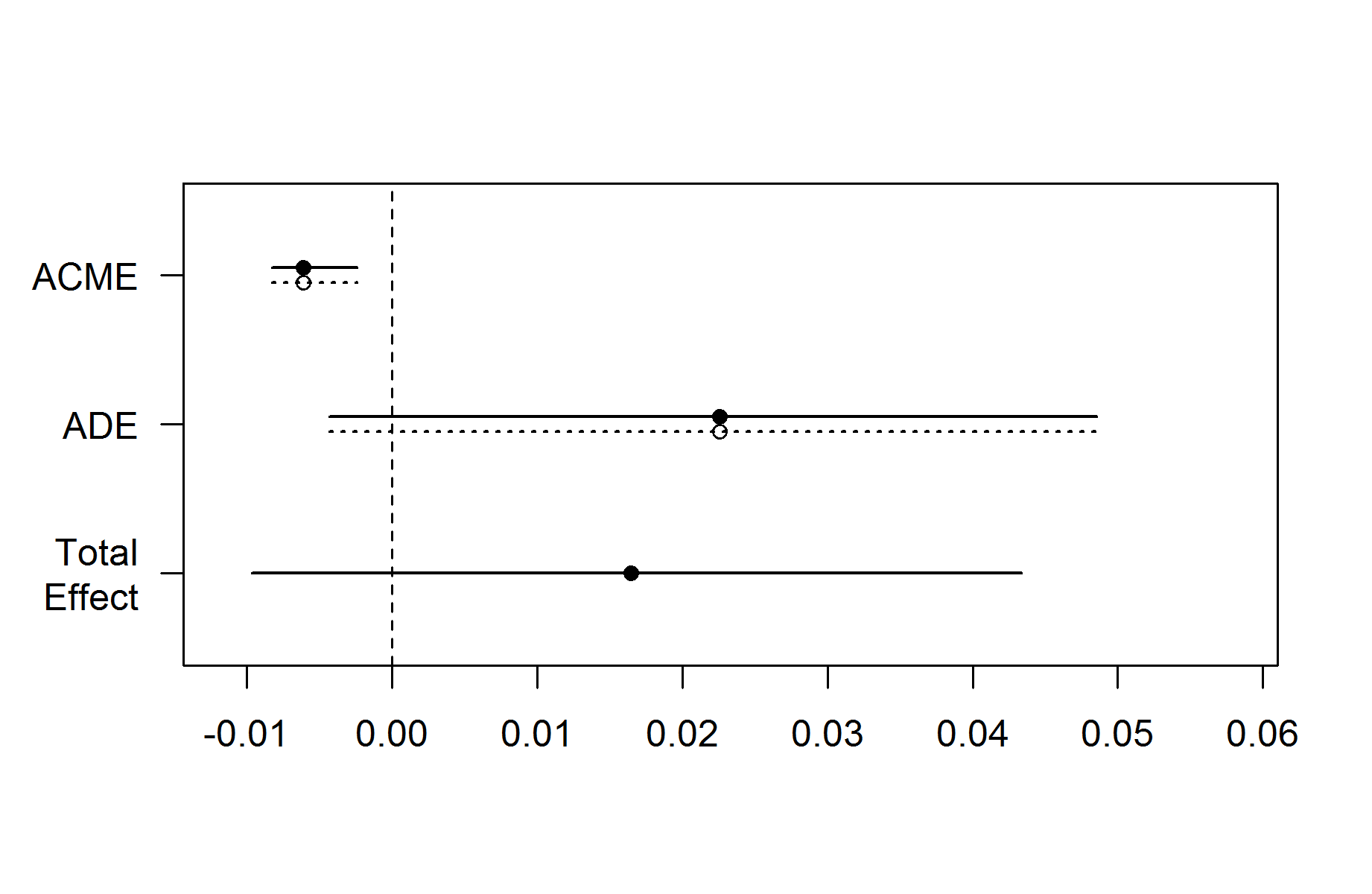


The mediation analysis suggested that sedation level [average causal mediation (ACME) effect: *b* = -0.0061; CI 95% -0.0061 to <-0.0001], but board-certified intensivists on morning and afternoon shifts were not [average direct effect (ADE): *b* = 0.0226; CI 95% -0.0043 to 0.05000].
